# Deep brain stimulation in patients on chronic antiplatelet or anticoagulation treatment

**DOI:** 10.1007/s00701-021-04931-y

**Published:** 2021-08-03

**Authors:** Joachim Runge, Luisa Cassini Ascencao, Christian Blahak, Thomas M. Kinfe, Christoph Schrader, Marc E. Wolf, Assel Saryyeva, Joachim K. Krauss

**Affiliations:** 1 Department of Neurosurgery, Hannover Medical School, Carl-Neuberg-Straße 1, 30625 Hannover, Germany; 2 Department of Neurosurgery, Universitätsmedizin Mannheim, Medical Faculty Mannheim, University of Heidelberg, Heidelberg, Germany; 3 Department of Neurology, Ortenau Klinikum Lahr-Ettenheim, Lahr, Germany; 4 Department of Neurosurgery, Division of Functional Neurosurgery and Stereotaxy, Friedrich-Alexander University, Erlangen-Nürnberg, Erlangen, Germany; 5 Department of Neurology, Hannover Medical School, Hannover, Germany; 6 Department of Neurology, Katharinenhospital Stuttgart, Stuttgart, Germany; 7 Center of Systems Neuroscience, Hannover, Germany

**Keywords:** Anticoagulation, Antiplatelet, Deep brain stimulation, Functional neurosurgery, NOAC, Vitamin K antagonist

## Abstract

**Background:**

In the aging society, many patients with movement disorders, pain syndromes, or psychiatric disorders who are candidates for deep brain stimulation (DBS) surgery suffer also from cardiovascular co-morbidities that require chronic antiplatelet or anticoagulation treatment. Because of a presumed increased risk of intracranial hemorrhage during or after surgery and limited knowledge about perioperative management, chronic antiplatelet or anticoagulation treatment often has been considered a relative contraindication for DBS. Here, we evaluate whether or not there is an increased risk for intracranial hemorrhage or thromboembolic complications in patients on chronic treatment (paused for surgery or bridged with subcutaneous heparin) as compared to those without.

**Methods:**

Out of a series of 465 patients undergoing functional stereotactic neurosurgery, 34 patients were identified who were on chronic treatment before and after receiving DBS. In patients with antiplatelet treatment, medication was stopped in the perioperative period. In patients with vitamin K antagonists or novel oral anticoagulants (NOACs), heparin was used for bridging. All patients had postoperative stereotactic CT scans, and were followed up for 1 year after surgery.

**Results:**

In patients on chronic antiplatelet or anticoagulation treatment, intracranial hemorrhage occurred in 2/34 (5.9%) DBS surgeries, whereas the rate of intracranial hemorrhage was 15/431 (3.5%) in those without, which was statistically not significant. Implantable pulse generator pocket hematomas were seen in 2/34 (5.9%) surgeries in patients on chronic treatment and in 4/426 (0.9%) without. There were only 2 instances of thromboembolic complications which both occurred in patients without chronic treatment. There were no hemorrhagic complications during follow-up for 1 year.

**Conclusions:**

DBS surgery in patients on chronic antiplatelet or anticoagulation treatment is feasible. Also, there was no increased risk of hemorrhage in the first year of follow-up after DBS surgery. Appropriate patient selection and standardized perioperative management are necessary to reduce the risk of intracranial hemorrhage and thromboembolic complications.

## Introduction

The management of chronic antiplatelet or anticoagulation treatment is a common problem in patients considered for elective surgery. Perioperative discontinuation of antiplatelet or anticoagulation treatment may increase the risk of thromboembolic and cardiovascular events [3, 8, 13] while continued treatment increases the risk of hemorrhage [23]. Particularly, in cerebral surgery, the risk of intracranial hemorrhage is significantly increased, making antiplatelet or anticoagulation treatment a relative contraindication for neurosurgical procedures [34]. Also, the timing of discontinuation of medication, the perioperative bridging of anticoagulation, and its resumption after neurosurgery are a matter of ongoing debate [21, 34].

Deep brain stimulation (DBS) with chronically implanted electrodes and implantable pulse generators (IPG) has become a common and widely accepted therapy for various movement disorders, chronic pain, and psychiatric disorders [14, 17, 19, 22]. Intracranial hemorrhage has been considered the most serious complication of DBS resulting in prolonged hospitalization, additional surgery, or permanent disability [20, 24, 29, 35]. While the frequency of asymptomatic hemorrhage ranges between 0.2 and 3.7%, symptomatic hemorrhage occurs in 0.0–1.6% of patients with an overall mortality of 0.0–0.7% [11, 38]. Therefore, there is an urgent clinical need to prevent hemorrhage and to develop strategies for its avoidance.

Perioperative management is crucial for an elective procedure such as DBS. There is an increasing number of elderly patients with movement disorders or chronic pain syndromes who often suffer also from co-morbidities such as cardiovascular diseases which require lifelong antiplatelet or anticoagulation treatment [4].

In general, any form of chronic anticoagulation treatment has been considered a relative contraindication for DBS [4, 11]. The question, however, arises whether an established therapy such as DBS can be denied to these groups of patients. While many neurosurgeons worldwide perform DBS in patients with chronic antiplatelet or anticoagulation treatment, there is very limited published data addressing this problem [4, 25, 31, 33, 36]. Furthermore, there are different drugs for antiplatelet or anticoagulation treatment, each with distinctive mechanisms and duration of action.

Here, we report our experience on the perioperative management of chronic antiplatelet or anticoagulation treatment in a series of 34 DBS patients and focus on the occurrence of hemorrhagic or thromboembolic complications with a follow-up for 1 year.

## Materials and methods

Between 2005 and 2018, 465 patients underwent functional stereotactic surgeries (DBS or radiofrequency lesioning) at Hannover Medical School, performed or supervised by the senior neurosurgeon (JKK). Indications were Parkinson’s disease (PD), dystonia, tremor, chronic pain syndromes, and psychiatric disorders. Results on clinical outcome and neurophysiology have been published in detail elsewhere [7, 9, 27, 28, 32].

Inclusion criteria for the present study were (1) chronic antiplatelet or anticoagulation treatment prior to and continued after functional stereotactic surgery; (2) implantation of DBS electrodes and an implantable pulse generator (IPG) for chronic stimulation. Exclusion criteria were transient test stimulation only via the DBS electrodes or radiofrequency lesioning.

For the present study, a retrospective analysis of the prospectively collected data was performed. All hemorrhagic and thromboembolic complications (intracranial and IPG pocket hematoma) were recorded during the perioperative period and for follow-up for 1 year. The patients were divided into three subgroups based on therapy regimes: antiplatelet drugs, vitamin K antagonists, and novel oral anticoagulants (NOACs). The subgroups were compared to each other and with the larger collective of patients without chronic antiplatelet or anticoagulation medication.

All patients with antiplatelet or anticoagulation treatment were first seen as outpatients. Patients were instructed to consult their general physician to assess their individual risk of transient withdrawal of treatment. Thereafter, they were instructed routinely how to proceed with treatment prior to the planned day of admission based on the different therapy regimes. Preoperative standard examination included an extensive anesthesiological work-up. Antiplatelet medication, such as aspirin or clopidogrel, regardless if used for primary or secondary prophylaxis, was stopped 7–10 days preoperatively. No bridging with high or low molecular weight heparin was initiated in these cases. The patients with vitamin K antagonists were instructed to stop medication and start bridging with subcutaneous low molecular weight heparin as soon as the international normalization ratio (INR) was below 2 before being admitted for surgery [23]. Patients with NOACs discontinued medication 2–3 days before surgery (approximately 5 half-lives) and bridged also with subcutaneous low molecular weight heparin. All patients gave informed consent to the planned procedure.

On the day of admission, coagulation lab values were determined including the INR and partial thromboplastin time (PTT). It was considered safe to perform DBS surgery with an INR < 1.2 (Quick > 80%), a PTT < 36 s, and a platelet count > 100.000. When used for bridging, fractionated heparin was stopped > 12 h prior to surgery, and continued 24 h after surgery.

The operative technique has been described in detail elsewhere [1, 9, 30]. Preoperative MRI scans were obtained in all patients. A Riechert-Mundinger stereotactic frame with the Zamorano-Duchovny semi-arc was used in all surgeries. Target and trajectory planning was performed on different work stations (Medtronic® StealthStation 3, StealthStation 7, Brainlab®) based on stereotactic 1.25-mm axial CT scans.

Patients were operated under local anesthesia via precoronal burr holes. Transventricular approaches were accepted. When the globus pallidus internus (GPi) or the subthalamic nucleus (STN) were targeted, microelectrode recording was performed as described previously [1, 30]. Intraoperative macrostimulation was achieved via the DBS electrodes to determine thresholds for effects and side effects. Standard quadripolar DBS electrodes were implanted via a guiding-cannula (Medtronic® 3387 or 3389; Boston Scientific® Vercise DBS Lead or Versice Cartesia Directional Lead) and used for chronic stimulation.

Stereotactic CTs were obtained immediately postoperatively in all patients to verify the electrode position and to detect any intracranial complications. The IPG was implanted under general anesthesia directly thereafter during the same operative session or within the following days.

Subcutaneous weight-adapted fractionated heparin was commenced 24 h postoperatively in all patients of the three subgroups for prophylaxis of thrombosis and to continue bridging. Intravenous antibiotics were administered for 48 h. Programming of the IPG was started after monopolar review and impedance measurements on the day after implantation of the IPG. Stitches were removed between days 10 and 12 after surgery. Bridging therapy with heparin was continued for 2 weeks after surgery. Thereafter, permanent antiplatelet or anticoagulation treatment was resumed and adjusted by the patients´ general physicians.

The Ethical Commission of Hannover Medical School indicated that no formal approval was needed for the present study.

For statistical analysis, the chi-square test (Fischer’s exact test) was used. Statistical significance was determined as *p* < 0.05.

## Results

Out of the total cohort of 465 patients, 34 patients (7.3%) were identified who were under chronic antiplatelet or anticoagulation treatment according to our inclusion and exclusion criteria. There were 24 men and 10 women, and mean age at surgery was 68.1 years (range ± 11.6). Out of these 34, 18 were on antiplatelet therapy, 13 had vitamin K antagonists, and 3 had NOACs. Demographical data, diagnoses, and DBS targets are shown in Table 1.

**Table 1:** Demographic data, diagnoses, and DBS targets in 34 patients

	Antiplatelet drugs	Vitamin K antagonists	NOACs
Total	18	13	3
Gender			
Male Female	12 6	10 3	2 1
Age (years)	67.1 ± 10.3	67.6 ± 13.6	76 ± 2.8
Diagnosis			
PD ET Dystonia Pain	7 5 5 1	1 10 2	1 2
DBS target			
STN	4	1	
Vim GPi	5 6	10 2	3
GPi + Vim CM-Pf + VPL	2 1		

*CM-Pf*, centromedian-parafascicular complex; *DBS*, deep brain stimulation; *ET*, essential tremor; *GPi*, globus pallidus internus; *NOAC*, novel oral anticoagulants; *PD*, Parkinson disease; *STN*, subthalamic nucleus; *Vim*, ventral intermediate nucleus; *VPL*, ventral posterior lateral nucleus

The group of patients with long-term antiplatelet therapy consisted of 18 patients (12 men, 6 women, mean age 67.1 ± 10.3 years). The indications for DBS were as follows: PD (7 patients)—4 bilateral STN, 1 bilateral Vim, 2 bilateral GPi; essential tremor (ET) (5 patients)—4 bilateral ventral intermediate (Vim) thalamic nucleus, 1 unilateral Vim + GPi; dystonia (5 patients)—4 bilateral GPi, 1 bilateral GPi + Vim; and chronic pain (1 patient)—unilateral centromedian-parafascicular complex (CM-Pf) and ventral posterior lateral (VPL) nucleus.

The group of patients with vitamin K antagonist therapy consisted of 13 patients (10 men, 3 women, mean age 67.6 ± 13.6). The indications for DBS were PD (1 patient)—bilateral STN; ET (10 patients)—10 bilateral Vim; dystonia (2 patients)—2 bilateral GPi. In the NOAC medication subgroup, there were 3 patients (2 men, 1 woman, mean age 76 ± 2.8 years). The indications for DBS were PD (1 patient)—bilateral Vim; and ET (2 patients)—2 bilateral Vim.

The most common indications for chronic antiplatelet or anticoagulation treatment were atrial fibrillation and coronary artery disease. Details are shown in Table 2. Three patients had multiple diagnoses which required combined treatment.

**Table 2:** Chronic antiplatelet or anticoagulant treatment in 34 DBS patients

	Antiplatelet drugs	Vitamin K antagonists	NOACs
Cardiac diseases			
Atrial fibrillation Coronary artery disease Mechanical valve	4 9 -	6 - 3	3 1 -
Cerebrovascular disorders			
Previous stroke Carotid stenosis	4 2	2 1	- -
Factor V thrombophilia	-	2	-

Treatment had been established by the patients’ general physician, internists, or cardiologists. Three patients had multiple disorders and required combined treatment (*n* = 37)

There were no intraoperative adverse events or complications, except in one patient who suffered from mild intraoperative air embolism with coughing, but without dyspnea or hemodynamic instability.

The postoperative stereotactic CT scan detected primary asymptomatic hemorrhage in 2/34 patients (5.9%). A 74-year-old man with tremor-dominant PD and long-term treatment with apixaban because of atrial fibrillation had an intraventricular hemorrhage associated with a transventricular electrode in the postoperative CT. After four asymptomatic days, his condition worsened due to hydrocephalus, requiring an external ventricular drainage for 5 days. At 3-month follow-up, hydrocephalus was no longer present and the tremor was remarkably improved under chronic DBS. A 39-year-old woman with unilateral tremor and aspirin prophylaxis because of a previous stroke had a small, asymptomatic intracerebral bleeding along the electrode trajectory in the postoperative CT scan. There were no long-term consequences.

The number of intracranial hemorrhages on the postoperative CT scan in patients without chronic antiplatelet or anticoagulation treatment was 15/431 (3.5%) which was not significantly different from the study group (*p* = 0.36, see Table 3).

**Table 3:** Intracranial hemorrhage, IPG pocket hematoma, and thromboembolic complications in patients without or on chronic antiplatelet or anticoagulation treatment

	Chronic antiplatelet or anticoagulation treatment (*n* = 34)	No chronic antiplatelet or anticoagulation treatment (*n* = 431)	Statistical significance
Intracranial hemorrhage	2 (5.9%)	15 (3.5%)	
No intracranial hemorrhage	32 (94.1%)	416 (96.5%)	n.s. (*p* = .36)
IPG pocket hematoma	2 (5.9%)	4 (1%)	
No IPG pocket hematoma	32 (94.1%)	417 (99%)	n.s. (*p* = .07)
Thromboembolic complication	0 (0%)	2 (0.5%)	
No thromboembolic complication	34 (100%)	429 (99.5%)	n.s. (*p* = 1)

Arterial hypertension was present in 16/34 (47.1%) of patients on chronic antiplatelet or anticoagulation treatment, and in 77/432 (17.9%) without. Intracranial hemorrhage occurred only in 2/93 patients with arterial hypertension (2.2%) and did not present as an independent risk factor in our series.

IPG pocket hematomas occurred in 2/34 patients (5.9%) in the group of patients on chronic antiplatelet or anticoagulation treatment. These patients had chronic medication with vitamin K antagonists. In the group of patients without antiplatelet or anticoagulation treatment, there were 4/421 (1%) IPG pocket hematomas. Note that 10 patients were excluded from this analysis as they either had radiofrequency lesions or no IPG was implanted. When comparing the two groups, there was no statistical significance (*p* = 0.07, see Table 3).

No thromboembolic or thrombotic complications occurred in patients on antiplatelet or anticoagulation treatment during the 30-day postoperative period, while such complications were noticed, however, in 2/431 patients (0.5%) who were not on chronic treatment.

During follow-up until 12 months after surgery, no additional thromboembolic or thrombotic or hemorrhagic complications occurred in the 34 patients on chronic antiplatelet or anticoagulation treatment. In particular, there were no instances of chronic subdural hematoma.

## Discussion

DBS surgery has become a widespread and accepted therapy worldwide with an estimate of more than 180,000 patients that have been operated [19]. Along with its application to new indications, in particular considering psychiatric disorders [19], there is also a continuous process of reevaluation of what should be considered a relative contraindication for DBS. While decades ago, for example, very young age or advanced age was considered red flags, there are now published studies on DBS for childhood dystonia in infants [37] or ET in geriatric patients [16]. Moreover, psychiatric disorders and dementia, formerly considered contraindications for DBS surgery, have now increasingly moved into focus [19]. Another example is the concomitant use of cardiac pacemakers and DBS neurostimulation systems. While initially it was thought that the risk of a concomitant use would be prohibitive, this practice has become accepted over the years given certain precautions being taken [10, 15].

In the present study, we show that DBS can be performed in patients on various forms of chronic antiplatelet or anticoagulation treatment with a reasonable risk/safety profile. We did not find an increased occurrence of hemorrhagic or thromboembolic/thrombotic complications after stopping treatment and providing perioperative bridging therapy, when indicated, in comparison to patients without chronic antiplatelet or anticoagulation treatment and between different types of drugs. The management of prophylactic treatment in the perioperative phase basically was in line with the proceedings used in other neurosurgical operations. There was also no increase of chronic hemorrhagic complications such as the development of subdural hematoma for up to 1-year follow-up.

The only patient who had a symptomatic hemorrhage in our present series was operated via a transventricular approach. There is disagreement whether such an approach might constitute per se an increased risk for hemorrhage or not [18, 26, 40].

The subject of our study has received only little attention thus far, despite the increasing age in the population and a more frequent use of antiplatelet or anticoagulant therapy [2]. In a previous study on 143 patients undergoing DBS surgery, no increased risk for intracranial hemorrhage was detected in 23 patients with previous use of anticoagulant medication [29]. The risk of IPG pocket hematoma and the occurrence of thromboembolic complications, however, were not evaluated and there was no differentiation between different forms of antiplatelet or anticoagulant therapies. Another study showed that postoperative prophylaxis with or without subcutaneous heparin for venous thromboembolism did not increase the risk for intracranial hemorrhage significantly [5]. More detailed analysis of the handling of chronic anticoagulation treatment in patients undergoing DBS or with existing DBS systems has been provided mainly in the form of case report or small case series [4, 25, 31, 33, 39]. We have reported previously on a patient who received bilateral pallidal DBS for severe chorea due to antiphospholipid antibody syndrome with a need for chronic phenprocoumon treatment. There were no complications of combined treatment at 2-year follow-up. In a series of 4 patients with DBS and chronic anticoagulation treatment, one IPG pocket hematoma occurred, but no intracranial hemorrhage [4].

The overall safety of DBS surgery in patients on chronic anticlotting therapy was also demonstrated in a recent study on a series of 226 patients of whom 90 patients were on chronic anticoagulation. There was no difference in adverse events between patients on anticlotting medication as compared to those without. Similarly like in our study, anticlotting treatment was paused before surgery, and a bridging protocol was installed when needed. Three hemorrhages occurred in the group who did not have chronic anticlotting treatment, and there was no instance of thromboembolic events within 90 days after surgery.

Intracranial hemorrhage in patients with DBS and subsequent anticoagulation therapy was reported very rarely. In one patient, high-dose intravenous heparin was administered on the first postoperative day for acute myocardial infarction [25], and in two other patients, edoxaban was restarted already on day 5 and day 7 after surgery [20, 33]. One of these patients also had a recurrent hemorrhage later. Delayed hemorrhage occurring 9 weeks after DBS surgery was described in a patient taking low dose aspirin, but this patient suffered also from arterial hypertension [39].

The incidence of IPG pocket hematoma was remarkably low in our study, although it was higher in patients on chronic antiplatelet or anticoagulation treatment than in those without. In this context, it is also of interest to review the practice of cardiac pacemaker surgery. One study showed that continued anticoagulation therapy with warfarin in pacemaker or defibrillator surgery actually reduced the incidence of clinically significant IPG pocket hematoma as compared to bridging therapy with subcutaneous heparin [6]. Another large prospective study on perioperative bridging of anticoagulation treatment with vitamin K antagonists in patients with atrial fibrillation showed that complete pausing of anticoagulation was noninferior to perioperative bridging with low molecular weight heparin for the prevention of thromboembolic complications and that it decreased the risk of major bleeding without causing more thromboembolic complications [12].

## Conclusion

We demonstrate the feasibility of implanting DBS systems in selected patients on chronic antiplatelet or anticoagulation treatment when taking certain precautions and we confirm its safety and efficiency over a follow-up period of 1 year.

## Abbreviations

DBS: Deep brain stimulation
NOACs: Novel oral anticoagulants
IPG: Implantable pulse generators
PD: Parkinson disease
INR: International normalization ratio
PTT: Partial thromboplastin time
GPi: Globus pallidus internus
STN: Subthalamic nucleus
ET: Essential tremor
Vim: Ventral intermediate nucleus
CM-Pf: Centromedian-parafascicular complex
VPL: Ventral posterior lateral nucleus
